# Technical note: A simple and effective CO_2_ delivery system for angiography using a blood bag

**DOI:** 10.4103/0971-3026.54881

**Published:** 2009-08

**Authors:** Mathew P Cherian, Pankaj Mehta, Prashant Gupta, Tejas M Kalyanpur, SR Jayesh, R Rupa

**Affiliations:** Department of Radiology, Kovai Medical Center and Hospital, Avinashi Road, Coimbatore, Tamil Nadu, India

**Keywords:** Carbon dioxide, CO_2_, contrast, DSA

## Abstract

Several angiographic techniques have been developed to image the arterial system, the commonest using iodinated contrast media. Useful as they may be, they are not without disadvantages. One other modality is angiography using CO_2_. Although CO_2_ can be used as an alternative contrast medium, delivery systems are expensive to procure. We describe an indigenous and effective delivery system developed at our institute.

## Introduction

Vascular diseases of the arterial system have long been a major cause of morbidity and mortality. Although several noninvasive techniques have evolved in recent times to image the arterial system, the gold standard remains the catheter digital subtraction angiography (DSA). Though the iodinated contrast media used today are relatively safe, they should, however, be used with caution in patients with compromised renal function and in diabetics. Patients with inadequate cardiac function are also poor candidates for angiography with iodinated contrast. Carbon dioxide (CO_2_) has a role in this subset of patients as an effective and safe contrast agent.[1]

Though gadolinium may be used as a substitute contrast agent[1] in patients with chronic renal failure, there is a chance of developing a potentially fatal complication called nephrogenic systemic fibrosis.[2,3]

CO_2_ has received immense attention in recent times for its excellent safety profile. There is a paucity of studies, related to procedures using CO_2_ in the Indian medical literature; a PubMed search in fact yielded no study from India regarding the use of CO_2_ in vascular procedures; this is probably due to the fact that dedicated delivery systems for CO_2_ are expensive to procure and maintain. This problem has been solved by developing an indigenous injection system at our institute.

## Materials and Methods

**Figure 1:**
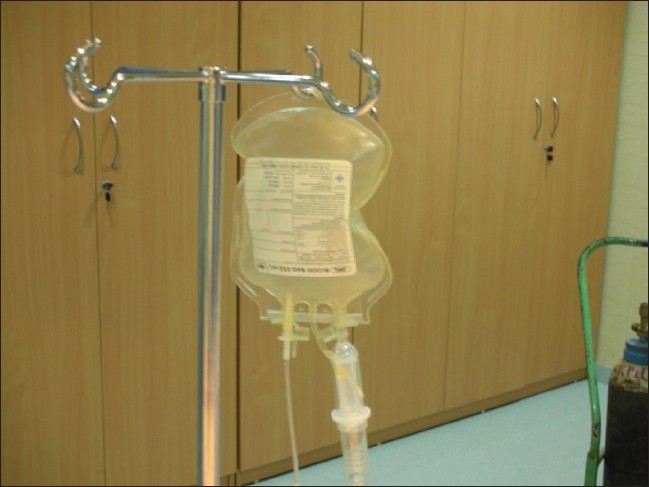
The blood bag inflated with CO_2_ is depicted

**Figure 2:**
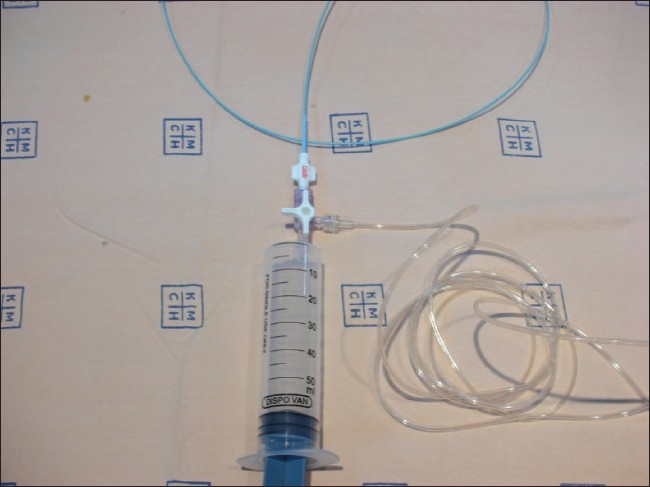
The syringe, three-way, and catheter assembly are shown

**Figure 3:**
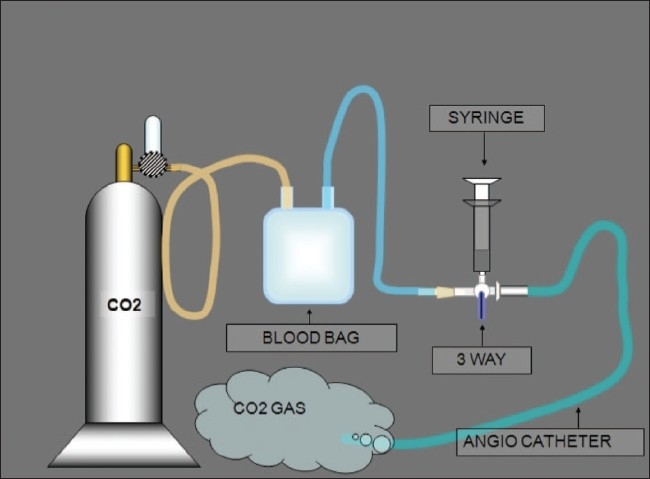
This schematic shows our delivery system

The system developed at our institute has been used for CO_2_ angiograms for many years now. It consists of a standard blood bag (readily available) connected to a CO_2_ cylinder [Figure 1]; this is done in order to avoid direct transmission of pressure from the cylinder to the vasculature under examination. The blood bag is then connected to a three-way stopcock [Figure 2], to which are connected a 50-cc plastic syringe and the angiographic catheter to be used [Figure 3].

**Figure 4:**
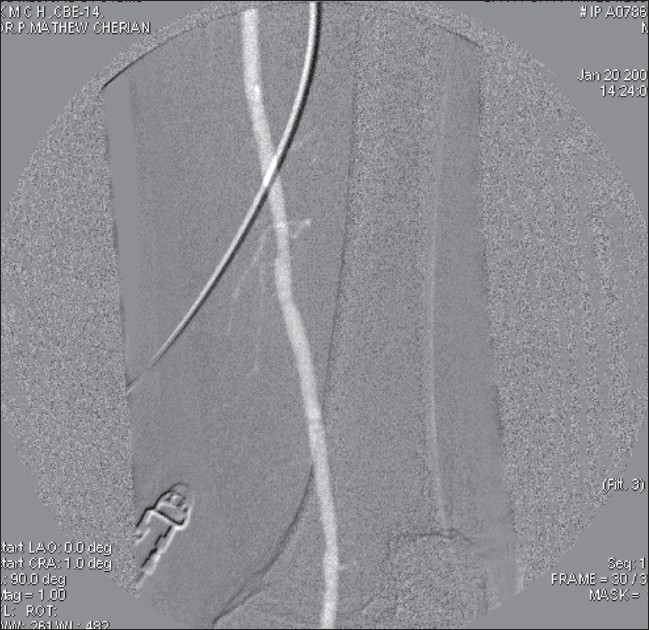
Angiogram with CO_2_ using stacking software shows the superficial femoral artery (arrow)

The blood bag is first filled with CO_2_ and the knob on the cylinder is closed. The bag now acts as a CO_2_ reservoir. CO_2_ is then allowed to freely flow for some time through the three ways in a basin containing sterile saline. This ensures that the tubings are free of air. The blood bag is intermittently filled to ensure that there is adequate CO_2_ in this low pressure reservoir. With the CO_2_ flowing through the ports of the three ways, a luer lock syringe is connected ensuring that the syring is kept upside down. A 50 cc syringe is used if the injection has to be made in the renal or infra renal aorta. We would use a 20 cc syringe in the iliac and femoral arteries. Seventy five percent of the syringe is filled with CO_2_ by gentle aspiration. The catheter is flushed with about 3 cc of CO_2_ to prevent explosive delivery of the agent. According to Caridi *et al*,[1] this explosive delivery is a major cause of patient discomfort. Once the catheter has been flushed, fifty percent of the CO_2_ in the syringe is injected to adequately visualize the vessel. The CO_2_ displaces blood in the vessel and provides ‘negative contrast’ [Figure 4]. The image quality can be enhanced by using ‘stacking software’. Stacking software is used because the CO_2_ column undergoes fragmentation as it travels distally; the software post-processes the images by stacking them one over the other, thus leading to an image in which the entire CO_2_ column is visualized (minimum intensity stacking software). This injection system was employed in a prospective study between September 2005 and August 2007, wherein 44 patients underwent lower limb arterial angiograms with CO_2_ and iodinated contrast (IC).

## Results

Of the 44 cases evaluated with this system, we obtained diagnostic quality angiograms in 42 (95.5%) cases. The system was easy to use and required minimal manpower and expertise to maintain and setup. There was no system malfunction at any time and no complications were encountered.

## Discussion

The commercially available delivery systems are not readily available in India and are difficult and expensive to procure. Hawkins *et al*,[4] developed a plastic bag system in 1995, which they later modified[5] to include O-rings to reduce the possibility of a CO_2_ leak; this system is commercially available as the Angioflush III. Our system has been indigenously developed and uses commonly available materials that are cheap to procure and maintain. The use of the blood bag as the reservoir avoids transmission of the high pressure of the gas in the cylinder to the vasculature; it also allows us to fill the syringe with a limited quantity of CO_2_. We circumvented the problem of CO_2_ leaks by using a three-way stopcock to which the catheter, syringe and the blood bag are attached. With proper operation of the stopcock, room air contamination can be completely avoided. This is an effective and cheap alternative to the commercially available CO_2_ delivery systems.
